# Suspicions regarding the genetic inheritance of acute lymphoblastic leukemia in patients with down syndrome

**DOI:** 10.34763/jmotherandchild.20222601.d-22-00002

**Published:** 2022-07-20

**Authors:** Emir Behluli, Nexhibe Nuhii, Thomas Liehr, Gazmend Temaj

**Affiliations:** Department of Pediatrics, University of Prishtina, Prishtina, Kosovo; State University of Tetovo, Faculty of Medical Sciences, Department of Pharmacy, Tetovo, North Macedonia; Institut für Humangenetik, Universitätsklinikum Jena, Friedrich Schiller Universität, Jena, Germany; Human Genetics, College UBT, Faculty of Pharmacy Prishtina, Kosovo

**Keywords:** Down syndrome (DS), acute lymphoblastic leukaemia (ALL), gene mutations, therapy

## Abstract

Children with Down syndrome (DS) are at markedly increased risk for acute lymphoblastic leukaemia (ALL). DS is caused by trisomy of chromosome 21 affecting approximately 1 in 732 newborns in the USA. ALL is the most common cancer in children and constitutes approximately 25% of cancer diagnoses among children under the age of 15. Different protocols for treatment and management of paediatric ALL are available; however, DS children with ALL (DS-ALL) have increased risk of therapy-related toxicity compared to those without DS. Herein, we summarize the available literature on inherited predisposition for ALL, and possibilities for molecular therapy and treatment for DS-ALL patients.

## Introduction

Acute lymphoblastic leukaemia (ALL) is a major paediatric cancer well-documented in Western countries [1]. Six decades ago, little to nothing was known about genetic factors being involved, especially in childhood ALL. However, thanks to many genetic studies including cytogenetic and molecular approaches, it has now been recognized that ALL consists of multiple subtypes, distinguishable by specific genetic lesion [2].

**Figure 1:**
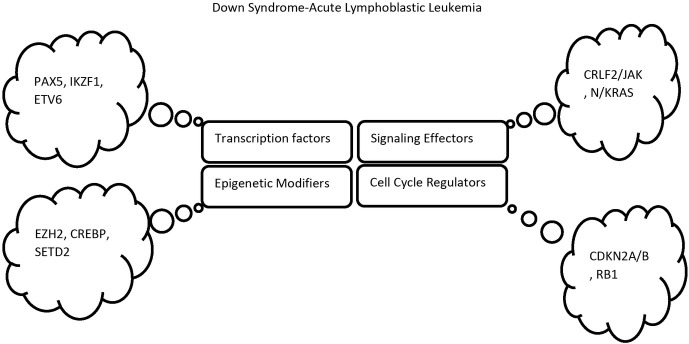
Somatic alterations found in DS associated with DS. Four most common types of association of DS with ALL in addition to constitutive trisomy 21.

Down syndrome (DS) (trisomy 21) is a chromosomal disorder affecting 1 in 732 newborns in the United States [3]. DS children have 20–50 times enhanced rates of developing ALL (DS-ALL) compared to children without DS [4,5]; especially for the subtype B-cell precursor ALL (BCP-ALL) [6]. Survival of DS-ALL compared to ALL patients without constitutional trisomy 21 is very poor [6]. In many studies of DS-ALL cases, mutations in the *GATA1* gene, a hematopoietic transcription factor, were detected [7]. Also, the following changes are typically found in DS-ALL: (i) *CRLF2* gene (essential lymphoid signalling receptor) overexpression [8], (ii) Janus Kinase 2 (*JAK2*) receptor mutations [8], (iii) somatic *IKZF1* deletions [9], (iv) hyperdiploidy [8, 10, 11, 12], (v) acquired *HMGN1* [13], (vi) *DYRK1A* [14], (vii) *PAX5* deletions [9], (viii) *ETV6-IGH* rearrangements [15], and (ix) less *ETV-RUNX1* gene fusion than in non-DS-ALL [5]. These alterations are discussed in more detail in the following sections and summarized in Figure 1.

## DS-ALL typical alterations

*CRLF2* (cytokine receptor like factor 2) is located in Xp22.33/ Yp11.32 in pseudoautosomal region 1 (PAR). Altered *CRLF2* (cytokine receptor like factor 2) gene expression in B-ALL, especially *CRLF2* overexpression, can be caused by deletion of PAR1, by translocations involving immunoglobulin heavy chain genes, e.g. IGH@-*CRLF2* and/ or *CRLF2* mutations (16). Deletion breakpoints arise typically between first non-coding exon *P2RY8* and coding region of *CRLF2* (12). Besides, in DS-ALL a point mutation in *CRLF2* gene resulting in F232C mutation has been observed [13, 16]. Mutations of *USP9X* gene (p.F1115Lfs) were found in 4 of 17 patients with *CRLF2* rearrangements as well [28].

In hematopoietic disorders *JAK2*-mutations like V617F are most common [17]. *JAK-STAT* has been shown to play a pivotal role in B-ALL; several studies demonstrate mutation of *JAK2*, particularly DS-ALL [18]. The *JAK2* mutations R683 and V617 disrupt the interaction of *JAK2* pseudokinase with the JH1/JH2 domains [22]. 19–28% of DS-ALL patients carry *JAK2* mutation R683 [19, 20, 21]. Also, *JAK2* gene mutations are reported, such as T875N, G861W, and for *JAK1* V658F and V617 (Table 1) [12]. Also, parallel gene mutations in *CRLF2* and *JAK2* are typical for B-ALL [12,16,25,26]. Associations between genes *JAK* and *CRLF2*, and *P2RY8-CRLF2* gene mutations were reported as well [12].

**Table 1:** Mutations in genes encountered in people with DS-ALL

Gene	Position of changes	References
JAK	V617F	[17]
JAK	p.R683G	[19]
JAK	p.R683K	[19]
JAK1	p.V658F	[12]
JAK2	p.T875N	[12]
JAK2	p.G861W	[12]
GATA3	c.778+1123T; c.779- 1748	[24]
PIP4K2A	c.678+761C>G	[24]
MSH6	p.T915A	[27]
IL7R	p.S185C	[28]
CRLF2	p.F232C	[28]
USP9X	p.F1115Lfs	[28]
PAX5	p.Gly186Ser	[30; 31]
IKZF1	p.Arg162Pro	[32]
IKZF1	p.His163Trp	[33]
IKZF1	p.Arg162Leu	[34]
IKZF1	p.Arg162Gln	[34]
NBN	p.R162W	[34]
NBN	p.K233Sfs*4	[34]
RTEL1	p.R918W	[34]
MLLT1	p.R473Q	[34]
FOXP1	p.Q3Sfs*80	[34]
ERG	c.373+951A>G	[4; 5]
CDKN2A	p.A148T	[37]
ETV6	p.A377T	[39]
ETV6	p.Y401N	[39]
ETV6	p.Pro214Leu	[40]
ETV6	p.Gln198*	[40]
ETV6	p.Leu379Pro	[40]
NF1	p.Val146Ile	[40]
RUNX1	p.Ile366_Gly367dup	[40]
ASXL1	p.Pro845Leu	[40]

*GATA3* is a transcription factor important in the differentiation of breast epithelia, urothelia, and a subset of T-lymphocytes [23]. The Varsome database shows intronic coding region mutations c.778+1123T>C and c.779-1748C>A for patients with ALL [24].

The gene *PIP4K2A* (phosphatidylinositol-5,4-bisphosphate), the precursor to second messengers of the phosphoinositide signal transduction pathways, is thought to be involved in the regulation of secretion, cell proliferation, differentiation, and motility. The protein belongs to a gene family capable of catalyzing the phosphorylation of phosphatidylinositol-5-phosphate on the fourth hydroxyl of the myo-inositol ring to form phosphatidylinositol-5,4-bisphosphate. In the patients with ALL mutations in the coding region of *PIP4K2A* these are reported as c.678+761C>G [24].

The *MSH6* protein is known to be member of the Mutant S family (MutS) which are involved in repair of damaged DNA. One single case of DS-ALL with mutation in this gene is reported also (p.T915A) [27]. SNV and insertion/deletion (indel) for *IL7R* (interleukin 7 receptor) and *CRLF2* are common in ALL patients, and Schwartzman et al. 2017 [28] has reported two DS-ALL patients with IL7R mutation (p.S185C) [28].

*PAX5* gene is member of the paired box family (*PAX*) of transcription factor (TF). The *PAX5* gene encodes the B-cell lineage-specific activator proteins (BSAP), a 52-kD molecule; *PAX5* is detectable only in B lineage and in early but not late stages of B-cell differentiation [29]. Two independent research groups have reported a specific *PAX5* gene mutation (p.Gly183Ser) in three pre-B ALL families with incomplete penetrance [30,31].

Putative functional germline variants in cancer-related genes (139 missense mutations; 3 frameshift deletions; and 1 splicing variant) were identified in 143 cases of ALL, especially the pathogenic variant p.Arg162Pro in *IKZF1* [32]. This heterozygote variant, together with an adjacent codon mutation as p.His163Trp, were recently reported as a pathogenic for patients with B-ALL [33]. Also, p.Arg162Leu could go together with p.Arg162G and lead to immunodeficiency and childhood B-ALL. Missense mutations (p.R162W) in patients with DS-ALL are reported also [34].

The *ERG* gene is located on chromosome 21 and mutations (as rs2836371 and c.673+951A>G) are associated with enhanced risk of DS-ALL [5].

The *CDKN2A-CDKN2B* locus in chromosome 9 is one of the most frequently deleted genomic regions in ALL patients [35,36]. In the *CDKN2A* (cyclin dependent kinase inhibitor 2A) gene missense mutation p.A148T is present in European descendent ALL patients [37].

## Genetic inheritance of ETV6 in patients with ALL

Besides the above-mentioned involvement of families with the *PAX5* gene mutation [30,31], one other gene in particular is observed to be mutated in ALL-families. Inherited thrombocytopenia and predisposition to developed haematological malignancies such as ALL were associated with pathogenic variants for *ETV6* (p.Pro214Leu, p.Gln198*) [38, 40] or (p.A377T, p.Y401N) [39]. *ETV-RUNX1* gene fusion is also a typical finding in ALL, thus, also a familial heterozygous *RUNX1* germline mutation (p.Ile366_Gly367dup) can lead to familial ALL [40].

## Treatment of DS-ALL patients

Liao and Liu [41] suggest that DS-ALL children’s treatment should be based on Dana Farber Cancer Institute Acute Lymphoblastic Leukemia Consortium protocols 00-001 (2000-2004) and 05-001 (2005-2011). Patients receive a multiagent remission induction consisting of weekly vincristine, prednisolone (40mg/m^2^/day for 28 days), L-asparaginase, and doxorubicin (total induction dose 60 mg/m^2^). In protocol 00-001, methotrexate (MTX) is administrated as a single high dose (iv 4g/m^2^) during induction; in protocol 05-001 MTX is administrated as a first low dose (40mg/m^2^) during first post induction phase, and after that as a second high dose (iv 5g/m^2^) during post induction phase [41].

Furthermore, Bohnstedt et al. [42] suggest treatment of patients according to protocols by the Nordic Society of Paediatric Haematology and Oncology (NOPHO) ALL92 (1992-2001) or ALL2000 protocol (2003-2007). The four-week therapy consists of prednisolone, vincristine, doxorubicin and intrathecal MTX application, followed by asparaginase. The oral therapy starts with a single dose of 6-mercaptopurine (6-MP) and MTX of 75 mg/m^2^ per day and 20 mg/m^2^ per week [42]. The first-year therapy for patients with SR (standard risk)-ALL and IR (intermediate risk)-ALL, consists of (i) VCR (vincristin) and glucocorticosteroids or (ii) high -dose MTX 5 g/m^2^ /24h with intrathecal MTX and leucovorin. In ALL2000 protocol, the 6MP, the starting dose is 50 mg/m^2^ per day applying thiopurine methylansferase for thiopurine methyltransferase heterozygous patients, and for completely deficient patients 5-10 mg/m^2^ [42]. Buittenkamp et al. [43] based their treatment on the Dutch Childhood Oncology Group (DCOG) for ALL treatment protocol [43]. The DS-ALL patient got a reduced or high dose of MTX, varying from 10% to 75% of the maximum dose, and intensified by leucovorin. The DS-ALL patients registered in the European Organization for Research and Treatment of Cancer (EORTC 58951) protocol from 2002 received 0.5 g/m^2^ of MTX instead 5 g/m^2^. DS-ALL patients treated by the Pediatric Oncology Group (POG 9405) protocol started with 50% of total dose of daunorubicin, cytarabine, teniposide, histone decaetylase, and PEG-asparaginase; this type of therapy showed reduction of toxicity [43]. Chessells et al. [44] treated based on 2 consecutive United Kingdom protocols (MRC UKALL X and XI) for ALL, consisting of daunorubicin, prednisolone, vincristine, MTX, and L-asparginase [44]. This included introduction treatment ([week 1–4] with daunorubicin, and on days 1 and 2, prednisolone, vincristine, intrathecal methotrexate, L-asparaginase), first intensification ([week 5– 8] treatment with daunorubicin, vincristine, cytarabine, etoposide, and thioguanine for 5 days), CNS directed therapy ([week 9–12] treated with cranial irradiation 18Gy and intrathecal methotrexate), and continuing treatment ([week 13–104] daily mercaptopurine, weekly methotrexate, monthly prednisolone and vincristine) [44]. In a study by Dördelmann et al. [45] treatment was according to BFM (Berlin-Frankfurt-Munster) protocols. The patients with DS-ALL were treated with methotrexate (MTX) during consolidation and prophylactic cranial irradiation (CRT) [45]. Matloub et al. [46] and Whitlock et al. [47] treated their patients according to the Children’s Cancer Group (CCG) protocol involving cytarabine, vincristine, dexamethasone, pegaspargase and MTX. Matloub et al. [46] used 5 doses of vincristine and escalating IV methotrexate (MTX) without leucovorin rescue in the interim maintenance (IM) phase: this gave superior event free survival (EFS) when compared with 2 doses of vincistrine, oral (PO) MTX, PO mercaptopurine and dexamethasone [46].

Kroll et al. [57] analysed MTX–associated toxicity during treatment with MTX (5 g/m^2^) plus intrathecal MTX and 6-MP consolidation therapy in patients with DS-ALL and non-DS-ALL enrolled in an ALL-Berlin-Frankfurt-Muenster (ALL-BFM) trial between 1995–2016 and 1995– 2007. From 2004 onwards a dose of 0.5 g/m^2^ of MTX was recommended for DS patients as those had higher rates of toxicities after the first treatment with 5 g/m^2^ MTX compared with non-DS-ALL patients. Higher MTX doses to 1.0 g/m^2^ did not result in an increased rate of toxicities after the second course in DS-ALL patients [57].

## Discussion

Children with DS have increased incidence for BCP-ALL during the first years of life [48]; the reason for that is still unknown. It is accepted that the presence of a constitutive trisomy of chromosome 21 is sufficient to disturb foetal haematopoiesis [49]. Partial or complete gains of chromosome 21 are frequently seen in non-DS children B-ALL cases, but very rarely are seen in adult leukaemia [50,51,52]. The observation shows that trisomy of chromosome 21 may prime the hematopoietic system for cancer and that DS-associated leukaemia could be used to study paediatric leukaemia in general.

Several studies show that increased phenotypic diversity and changes in selection dynamics in the foetal liver and bone marrow may have a role in leukemic development in non-DS and DS children. Generally the mutation landscape of childhood leukaemia is different from adult leukaemia [52,53]. Cancer driver mutations found in DS-associated leukaemia are less frequently found in non-DS-associated leukaemia [19,35,54, 55, 56].

In conclusion, in-depth knowledge of the inherited and somatic genetic alterations in ALL has provided a compelling rationale to harness precision medicine opportunities for paediatric ALL, from refining molecular diagnosis, identifying new prognostic biomarkers, incorporating molecularly targeted therapies, and introducing genetic-guided dose adjustment. Implementing genetic counselling and cancer surveillance is also helpful in patients with inherited cancer susceptibility.

Therefore, precision oncologic diagnostics in paediatric ALL is a good example for illustrating the power of personalized medicine. During the next decades it is necessary to focus on the new challenges of precision medicine, and to establish a strategy to translate new genomic discoveries into therapy for children suffering from ALL. Implementation of NGS into clinical laboratory routine diagnostics and development of cost-effective diagnostic platforms to provide access for all patients at diagnosis will be paramount. Molecular therapy in all subtypes of ALL will require international collaborations to design prospective protocols and methods for cure. Efforts will be focused on investigating the mechanisms of TKI and combination strategies or immunotherapy and small-molecule inhibitors.

## Abbreviations

ALL: acute lymphoblastic leukemia
DS: Down syndrome
DS-ALL: Down syndrome – acute lymphoblastic leukemia
BCP-ALL: B-cell precursor ALL
*GATA1*: gene for hematopoietic transcription factor
*CRLF2*: cytokine receptor-like factor 2
*JAK2*: Janus kinase 2
*IKZF1*: Ikaros family zinc finger protein 1
*HMGN1*: high mobility group nucleosome binding domain 1
*DYRK1A*: dual specificity tyrosine-phosphorylation-regulated kinase 1A
*PAX5*: paired box protein Pax-5
*ETV6-IGH*: translocation-Ets-leukemia virus 6- immunoglobulin heavy chain
*ETV-RUNX1*: Ets-leukemia virus- Runt-related transcription factor 1
*CRLF2*: cytokine receptor like factor 2
*PAR1*: a serine/threonine-protein kinase of the KIN2/PAR-1/MARK kinase family
JH1/JH2: domains of JAK2 pseudokinase
*GATA3*: transcription factor that in humans is encoded by the GATA3 gene
*PIP4K2A*: phosphatidylinositol-5,4-bisphosphate
*P2RY8*: P2Y purinoceptor 8
*MSH6*: protein, member of the Mutant S family (MutS)
SNV: single nucleotide variant
*IL7R*: interleukin 7 receptor
*USP9X*: Probable ubiquitin carboxyl-terminal hydrolase FAF-X
*BSAP*: B-cell lineage-specific activator proteins
*NBN gene*: nibrin gene
*MLLT1 gene*: myeloid/lymphoid or mixed-lineage leukemia; translocated to 1
*ERG gene*: ETS-related gene
*CDKN2A*: Homo sapiens cyclin dependent kinase inhibitor 2A
*CDKN2B*: Homo sapiens cyclin dependent kinase inhibitor 2B
*RUNX1*: Runt-related transcription factor 1
HD-ALL: high-hyperdiploid acute lymphoblastic leukemia
MTX: methotrexate
MP: mercaptopurine
BCP-ALL: B-cell precursor acute lymphoblastic leukemia
NGS: next generation sequencing
TKI: tyrosine kinase inhibitors
